# Feasibility, acceptability and validity of SMS text messaging for measuring change in depression during a randomised controlled trial

**DOI:** 10.1186/s12888-015-0456-3

**Published:** 2015-04-03

**Authors:** Stewart J Richmond, Ada Keding, Magdalene Hover, Rhian Gabe, Ben Cross, David Torgerson, Hugh MacPherson

**Affiliations:** 1 Sydera Research Associates, 34 Shipman Road, Market Weighton, York YO43 3RB UK; 2 Department of Health Sciences, University of York, Heslington, York YO10 5DD UK; 3 Department of Paediatrics, Hull and East Yorskshire Hospitals NHS Trust, Hull Royal Infirmary, Anlaby Road, Hull, HU3 2JZ UK

**Keywords:** Text messaging, SMS, Depression, Outcome assessment, Randomised controlled trial, Validity

## Abstract

**Background:**

Despite widespread popularity, text messaging has rarely been used for data collection in clinical research. This paper reports on the development, feasibility, acceptability, validity, and discriminant utility of a single item depression rating scale, delivered weekly via an automated SMS system, as part of a large randomised controlled trial.

**Methods:**

755 depressed patients (BDI-II score ≥20) were recruited from primary care into a randomised trial of acupuncture versus counselling or usual care, and invited to opt into a repeated-measures text messaging sub-study. Two weeks following random allocation, trial participants were sent a weekly text message for 15 weeks. Texts were a single question asking, on a scale from 1 to 9, the extent to which they felt depressed. Feasibility and acceptability of the automated SMS system were evaluated according to cost, ease of implementation, proportion consenting, response rates, and qualitative feedback. Concurrent validity was estimated by correlating SMS responses with the Patient Health Questionnaire (PHQ-9). SMS responses were compared between groups over time to explore treatment effects.

**Results:**

527 (69.8%) trial participants consented to the texting sub-study, of whom 498 (94.5%) responded to at least one message. Participants provided a valid response to an average of 12.5 messages. Invalid responses accounted for 1.1% of texts. The automated SMS system was quick to set-up, inexpensive, and well received. Comparison of PHQ-9 and SMS responses at 3 months demonstrated a moderate to high degree of agreement (Kendall’s tau-b = 0.57, *p* < 0.0001, n = 220). SMS depression scores over the 15 weeks differed significantly between trial arms (p = 0.007), with participants allocated to the acupuncture and counselling arms reporting improved depression outcomes compared to usual GP care alone, which reached statistical significance ten weeks after randomisation. Overall, the single item SMS scale also appeared more responsive to changes in depression, resulting from treatment, than the PHQ-9.

**Conclusions:**

Automated SMS systems offer a feasible and acceptable means of monitoring depression within clinical research. This study provides clear evidence to support the regular use of a simple SMS scale as a sensitive and valid outcome measure of depression within future randomised controlled trials.

**Trial registration:**

Current Controlled Trials - ISRCTN63787732

http://www.controlled-trials.com/ISRCTN63787732/ACUDEP

Date of registration: 15/12/2009

## Background

Sending and receiving text messages, via ‘short message service’ (SMS), is reported to have become the most frequently used method of communication between family and friends in the UK, with an average of 1.3 mobile telephones in use for every UK adult, and an average of 200 texts sent per month per user [1,2]. Over recent years, automated SMS systems have also become widely established for both commercial and non-commercial purposes. These systems link in with databases containing contact details to enable text messages to be sent en masse to multiple mobile phone users at pre-specified times, and elicit a reply if required. Yet, despite the popularity of text messaging as a quick and affordable method of communication, and the extensive use of automated SMS systems, there has been very limited research to explore the potential applications and benefits of automated text messaging for clinical research purposes. Here we distinguish such applications from the paradigm of ecological momentary assessment, which involves far more intensive real time data capture [3].

Research on the use of text messaging for the collection of clinical data appears to have focused largely on monitoring lower back pain [4-9]. In these studies participants were asked to reply to text messages, sent out on a weekly basis, by reporting on either the number of days they had been bothered by back pain, time taken off work, or by providing a single symptom score for their back pain on that particular day. Pilot studies and small scale trials have also investigated the possible use of text messaging for the purposes of monitoring and data collection for other areas of clinical interest, including: sexual health [10]; schizophrenia [11]; bulimia nervosa [12]; asthma [13]; alcohol rehabilitation [14]; and patient satisfaction [15]. One recent study employed a two way SMS system in which participants with rheumatoid arthritis completed the EQ-5D quality of life measure, by responding to multiple text messages, each corresponding to a different item, sent at one minute intervals [16].

Compared with traditional approaches, e.g. involving postal questionnaires, the application of text messaging as a method for gathering self-report outcome data in clinical trials may confer a number of advantages. Text messaging has been found to represent a relatively inexpensive means of collecting data and patients commonly reply in a timely manner [5,10,14,16]. Text messaging may also be less burdensome, because participants can be reached easily, and can respond quickly, wherever they are. This offers the possibility of monitoring participants on a more frequent basis outside of a controlled research environment, which may be especially useful in plotting symptoms over time to determine the optimal duration or frequency of treatments in terms of their efficacy or cost-effectiveness.

Findings from previous research suggest that trial participants find text messaging an acceptable method of data collection, although response rates vary [9-11,13,14,16]. Participants involved in a recent study concerning schizophrenia also expressed concerns that reliance on simple symptom scores derived from text messages might inadequately represent their experiences, suggesting that they should accompany other more traditional measurement and assessment protocols [11]. Indeed, whilst automated systems may provide an opportunity to gather large volumes of data from many recipients in a quick and cost-effective manner, one obvious and practical disadvantage regarding a standard text message is that it is limited in length, to phrases comprising less than 160 characters. Formatting restrictions also hinder the presentation and collection of complex information, for which printed questionnaires or diaries may be better suited. Nevertheless, text messaging may serve as a useful adjunct to more traditional methods of data collection until MMS (multimedia messaging service) and smartphone use becomes cheaper and more widespread.

Text messaging may further alleviate problems of incomplete data, threatening both internal and external validity in clinical research. This poses a particular problem for research involving groups of people who may be less inclined to respond to postal requests or attend appointments, e.g. patients with depression. In such cases, simple text responses could provide valuable supplementary information which might be used to impute missing data gathered by more conventional methods. Moreover, automated SMS systems can also be used to improve data collection by reminding research participants to attend appointments, return questionnaires, etc.

Research on the use of text messaging for collecting outcome data on experiences of depression is extremely limited, and has not been attempted in RCTs. A search of PubMed from inception to 19^th^ March 2014 using the terms ‘sms’ and ‘depression’, and ‘text messaging’ and ‘depression’, revealed only 70 published papers, of which just three reported original findings on the use of text messaging as an outcome measure for low mood or depression. Two of these studies concerned the use of weekly text messages amongst participants with bipolar disorder to plot the longitudinal course of the disorder [17] and mood forecasting [18]. The other small case study investigated the feasibility of daily text messaging to monitor mood among patients with anxiety and depression in a remote Australian community [19]. The latter used a 0 to 10 rating scale, and was found to be easy to implement, resulting in good compliance, and valuable clinical data.

Peer review of the present manuscript in June 2014 identified a further two relevant research articles, which were published in journals not listed by PubMed. The first of these describes a commercially available SMS instrument (*Mood 24/7*) for monitoring mood [20]. Whilst this study reported good daily compliance (87%) in use amongst a non-clinical population, the instrument described does not yet appear to have been validated for use as an outcome measure amongst patients with depression. The second paper describes an exploratory study using text messaging amongst twelve, English or Spanish speaking, patients enrolled into a group based cognitive behavioural therapy programme [21]. This included messages asking participants to report their current mood on a scale from 0 to 10. Results from this study indicate that text messaging may provide a useful low-cost means of improving engagement and attendance for group psychotherapy. The average response rate to text messages was 65%, although again, the SMS mood scale developed for this study was not compared to any previously established outcome measure of depression.

The present study examines the feasibility, acceptability, validity and utility of SMS text messaging as a method of collecting repeated self-rated data on experienced depression from participants in a randomised controlled trial. The study describes the development and concurrent validity of the 9-point SMS depression rating scale adopted by this study in relation to other established patient reported outcome measures used in evaluations of treatment for depression.

## Methods

### Design

A text messaging sub-study was incorporated within a randomised controlled trial investigating the therapeutic effects of acupuncture plus usual GP care and counselling plus usual GP care compared to usual GP care alone. This used a repeated-measures design, beginning immediately prior to the start of a twelve week trial intervention period. Participants were invited to respond to SMS text messages sent out weekly, over a period of fifteen consecutive weeks, which asked participants to rate their experience of depression on a simple 9-point scale, worded to capture a subjective aggregate for the prior week.

### Participants

755 patients were recruited from 27 general medical practices located across Northern England to take part in a randomised controlled trial, referred to as the ACUDep trial (ISRCTN63787732), which aimed to compare the effects of acupuncture, counselling, and usual GP care for managing depression [22]. All participants were 18 years of age or older, had consulted for depression within the previous five years, and had a score of 20 or above at baseline on the Beck Depression Inventory (BDI-II), which this scale classed as ‘moderate’ or ‘severe’ depression [23]. Participants were randomly allocated to acupuncture, counselling and usual care with a ratio of 2:2:1 respectively. Those recruited into the trial were also invited to take part in an optional sub-study involving the use of weekly SMS text messages to monitor depression.

### Development of a simple SMS depression rating scale

A panel of five people from the Department of Health Sciences at the University of York was formed to determine the most appropriate method of collecting clinical data on depression by means of text messaging. The panel comprised members with a broad range of expertise including health research, general medical practice, psychology, nursing, psychometrics, and data management.

The panel initially agreed on: (1) the use of SMS rather than multimedia messaging service (MMS) texts, for reasons of cost and compatibility with older mobile telephones; (2) the importance of minimising the burden on respondents, by presenting a brief and widely intelligible question with a single digit response format; and (3) the ideal frequency and period over which texts would be sent.

Our aim was to devise a direct and easy to comprehend question that would encompass a broad spectrum of individual experiences relating to depression. Wording of the item involved an initial brainstorming session to generate many potentially suitable items. The panel then engaged in an iterative process shortlisting items, and discussion of precise wording, finally reaching consensus on the use of a single question, which was worded as follows:

*ACUDep Trial: Over the last week how depressed have you felt on average? Please reply with a score between 1 and 9; where 1 is “not at all” and 9 is “extremely”*

Richmond et al. Short Message Service Depression Scale (R-SMS-DS)

### Data collection

Text messages were sent out weekly over a period of fifteen consecutive weeks, beginning two weeks after randomisation. This allowed time to organise and send out appointment letters to participants who were randomly allocated to receive acupuncture or counselling followed by attending up to twelve weekly sessions. The text messaging study thereby covered the normal trial intervention period.

Trial participants who agreed to take part in the text messaging sub-study and provided their mobile telephone number were sent a £5 note at the outset of the study, via post, to cover in advance all reasonable cost of equipment (i.e. use of their mobile telephone) and replying to text messages.

The research team chose SMS Gateway services provided by *IntelliSoftware Ltd.*, as a platform for text message automation [24]. This linked in with a Microsoft Access database, which generated reminders to initiate the distribution of texts. Texts were sent out on Thursdays at 12.30 pm. Thursdays were chosen because this is when study randomisation normally occurred, so the first text went out exactly two weeks after entry into the trial. The timing of texts at mid-day aimed to coincide with lunch, when people would be taking a break from work, to increase the probability of an immediate response.

Participants also completed paper questionnaires as part of the main ACUDep trial, which included established outcome measures of depression; the BDI-II (at baseline and 12 months) and the PHQ-9 (at baseline, 3 months and 12 months after randomisation) [25]. The BDI-II contains 21 questions; each answer being scored in the range 0 to 3, so overall scores can range from 0 to 63 with higher scores indicating more severe depression. The cut-offs are 14, 20 and 29 for mild, moderate and severe depression. The PHQ-9 is a nine item depression scale. Each item is scored between 0 and 3, thus PHQ-9 scores can range from 0 to 27 with higher scores indicating greater depression. In practice scores of 5, 10, 15, 20 have been used as cut points for mild, moderate, moderately severe and severe depression. For both the BDI-II and PHQ-9 respondents are asked to report how they have been feeling over the preceding two weeks.

### Validation of SMS scores

All texts sent to and received from participants were collated in an Excel spread sheet and exported into Stata (Version 12.1) for analysis. Received texts were matched to texts sent according to date. Texts received from participants were considered valid if they contained a single numeric or alphanumeric depression score between 1 and 9, either by itself or included in additional narrative. Half scores were also allowed, or derived if two adjacent scores were given, and included in the analysis. If participants explicitly corrected a previously submitted score on the same day, the updated score was used. If multiple texts were received in response to a sent message, only the first valid text response was kept for analysis.

### Analysis

#### Feasibility

The ease of implementation of the SMS system was summarised descriptively together with associated costs. Any technical problems and issues arising from using the SMS system in a population experiencing mental health problems were highlighted. The nature of any texts that could not be considered valid was explored.

#### Acceptability

Acceptability was evaluated in terms of consent and response rates. The number and percentage of participants responding to any text over the 15 week study period was summarised as well as the mean number of responses provided by these patients. Participants were also offered the opportunity to comment in their questionnaires about their experiences of taking part in the trial, which included the SMS sub-study.

#### Validity

The distribution and range of the SMS depression scores were investigated by descriptive statistics and changes explored over time. Whilst the first text messages were sent out two weeks after collection of PHQ-9 scores at baseline, the final depression score coincided with PHQ-9 assessment at 16 weeks. Concurrent validity of SMS scores was assessed against PHQ-9 depression at 16 weeks post randomisation using Kendall’s tau-b (p < .05). Tau-b was chosen to account for a large number of expected ties in the data. Only text messages received within +/−6 days of questionnaire completion at that point were included in the analysis. In order to evaluate which aspects of depression SMS responses predominantly related to, individual PHQ-9 items were also correlated with SMS scores. For comparative purposes, we investigated the degree of association between PHQ-9 and BDI-II scores at their concurrent data collection points at baseline and 12 months, again using Kendall’s tau-b statistic.

#### Utility

The primary ACUDep trial analysis showed a statistically significant reduction in PHQ-9 depression at three months for acupuncture (−2.5 score points, 95% CI: −3.7, -1.2) and counselling (−1.7 score points, 95% CI: −3.0, -0.5) compared to usual care. Details regarding the interventions and results are provided in the trial protocol and main results paper [22,26]. In order to evaluate the potential utility of SMS depression scores to detect the group differences over the same time period among those opting in to the SMS messaging, trajectories of change across the three ACUDep trial arms were analysed using a random slope linear mixed model. Texted depression scores over 15 weeks were predicted by trial arm, time and trial arm by time interaction, adjusting for baseline depression (PHQ-9). Time points were nested within patients. The statistical significance (p < .05) of the interaction term was used to identify whether the rate of change in reported depression differed between the intervention groups. Any significant interaction was further investigated by group contrasts at each time point. The analysis was carried out on an intention-to-treat basis. Sensitivity to change of the SMS scores was related to that of the PHQ-9 total and individual items by comparing differences in unadjusted standardised means at 3 months and resulting standard effect sizes.

### Ethical approval and consent

Full ethical approval for the trial was granted by York NHS Research Ethics Committee on 21^st^ September 2009 (ref: 09/H1311/75), together with research governance approval shortly thereafter from North Yorkshire & York Primary Care Trust. All participants provided informed written consent.

## Results

### Participants

**Figure 1:**
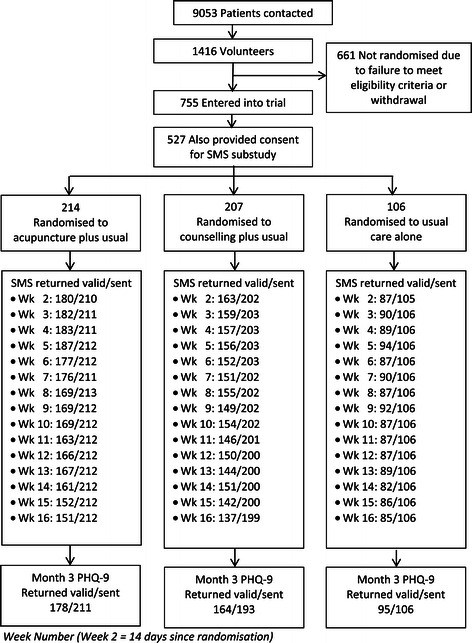
**Participant flowchart for SMS sub study.**

Patient recruitment began in December 2009 and finished in April 2011. Figure 1 illustrates the flow of patients through the SMS sub-study. 527 people (from a total of 755 trial participants) consented to taking part in the SMS sub-study. Baseline characteristics of patients who did and did not consent to the texting sub-study are presented in Table 1. Consenters tended to be younger, female, in employment, and reported experiencing their first major episode of depression at a younger age than those who declined to take part in the texting sub-study. However, levels of depression were comparable in terms of their BDI-II, PHQ-9 and EQ-5D anxiety/depression scores.

**Table 1:** **Baseline characteristics of patients consenting/not consenting to texting sub-study**

Characteristic	Consenting	Not consenting	Randomised
	n = 527 (69.8%)	n = 228 (30.2%)	n = 755 (100%)
Age: Mean (SD)			
Years	41.6 (11.5)	49.5 (15.6)	44.0 (13.4)
Gender			
Female	399 (75.7%)	155 (68.0%)	554 (73.4%)
Employment			
Working full-time	217 (42.3%)	64 (28.3%)	281 (38.0%)
Working part-time	104 (20.3%)	40 (17.7%)	144 (19.5%)
Unable to work	61 (11.9%)	34 (15.0%)	95 (12.9%)
Looking after home/family	62 (12.1%)	21 (9.3%)	83 (11.2%)
Retired	19 (3.7%)	46 (20.4%)	65 (8.8%)
Full-time education	21 (4.1%)	2 (0.9%)	23 (3.1%)
Other	29 (5.7%)	19 (8.4%)	48 (6.5%)
Total	513 (100%)	226 (100%)	739 (100%)
Depression: Mean (SD)			
Age at first major episode	23.7 (10.7)	28.5 (14.8)	25.2 (12.3)
BDI-II	32.6 (8.6)	32.4 (9.2)	32.6 (8.8)
PHQ-9*	16.0 (5.3)	15.8 (5.1)	16.0 (5.3)

*26 patients had 1 or 2 PHQ9 questions missing and therefore, their overall PHQ9 score was pro-rated.

### Feasibility

#### Ease of implementation

Set-up of the automated SMS system was achieved using an established in-house trial management database, built in Microsoft Access, which was linked to an online SMS platform. This generated text messages, sent individually to study participants, on pre-determined dates according to time since randomisation. This set-up process, of linking the management database and online SMS platform, took an experienced data manager just one day to complete. Incoming replies were then held in an online password protected system, which could be downloaded as .csv files. This system was generally very reliable. The majority of participants (507, 96.2%) were sent 15 weekly texts, while 20 participants (3.8%) were sent between 1 and 14 texts, which accounted for those participants who withdrew. Participants withdrew by notifying the research team and were not required to give a reason [22].

#### Technical problems

Whilst relatively straightforward to implement, one important technical problem was encountered with this system during its use. On the 28^th^ April 2011 research staff began receiving complaints from participants who were having difficulty replying to text messages. This issue took one week for the research team to investigate properly, at which point staff at *IntelliSoftware* acknowledged that there had been ‘bug’ in the system, which they had been aware of and corrected, but they had failed to notify all of the affected account holders. This error, apparently, involved the omission of ‘+’ symbols preceding telephone numbers contained within text messages, including some general reminders, which were sent to 113 trial participants. Given the time taken to diagnosing the problem, which could otherwise have been rectified within 24 hours, it is estimated that this error resulted in loss of clinical data from approximately 50 SMS responses. All participants concerned received an apology.

#### Nature of messages received

Responding patients submitted a total of 6,541 individual text messages, in response to 7,787 of sent texts. Of all text messages received, 6,137 (93.8%) were considered valid (single scores or extracted from additional narrative), 71 messages (1.1%) were invalid (out of range or not including score information), and 333 (5.1%) messages were additional responses to the same texts. Most of the extraction of valid scores was easily achieved by programmatic data manipulation. However, the categorisation of texts containing additional information required considerable manual inspection.

#### Incidences relating to participant welfare

Non-numerical responses received via the automated SMS system revealed serious welfare concerns regarding four participants during this study.

Early on in the trial, one participant issued a suicide threat via SMS, which read: *“I am going to kill myself. A decision which I found very very easy. More vodka first. Bye world.”* One week later, in reply to a second automatically generated text message, the following response was received: *“Please refrain from texting this number. The previous owner has passed away”,* an event we found later did not take place*.* Because no member of the research team had anticipated that the SMS system might be misused in this way, the content of these messages went unread for four weeks. During this period the gentleman concerned took part in an in-depth qualitative interview, in which he revealed that had sent these texts as *“a joke”*, after feeling disgruntled for being allocated to receive counselling rather than acupuncture (his preferred choice). This incident led to a review of all text messages received and the implementation of an active monitoring system, which raised immediate concerns regarding the welfare of a second participant.

The second incident involved a respondent who sent the research team a total of 161 non-numerical text messages over a space of just four weeks. These appeared increasingly unrelated to the trial and more bizarre. This led to a telephone conversation with the trial manager (SR), involvement of the participant’s GP, and an urgent referral to specialist mental health services, which confirmed that the participant was experiencing a psychotic episode.

Two further incidents involved the receipt of text messages which indicated an immediate risk of self-harm. In one case this led to input from a crisis resolution (emergency mental health) team, whereas no further action was taken in the other case, as the person revealed that he had sent the message whilst drunk and had no intention of harming himself.

#### Costs

Text messages sent via the SMS Gateway cost between 6 to 7 pence per SMS, depending on the number of ‘credits’ purchased. Given differences in response rates to the first and final text messages (83.1% and 72.1% respectively), this equates to a cost of between 8 to 9 pence for each of the 6137 valid SMS responses received. However, each trial participant who consented to the SMS sub-study also received £5 at the outset of the trial to reimburse SMS expenses. Given a total cost of £2,635, this extended the cost of text messaging to between 52 to 53 pence for every valid SMS response received. Other associated human resource costs were more difficult to estimate. Whilst development work to establish the automated SMS system only took our data manager one day, the introduction of regular monitoring of SMS responses proved more time consuming for research staff. Typically this activity took the trial support officer between one to two hours per week, and on occasions involved further input from the trial manager.

### Acceptability

#### Consent and response rates

69.9% (527/755) of participants in the main trial agreed to take part in the SMS sub-study. Since consent was given prior to randomisation, the proportions of trial participants also taking part in the SMS sub-study was roughly equivalent between treatment arms (Acupuncture = 70.9%; Counselling = 68.5%; Usual Care = 70.2%). No reasons were given for refusing to opt into the SMS sub-study, although many of the participants who declined also failed to provide a mobile telephone number in the contact details section of their trial consent forms.

Of the 527 consenting patients, 498 (94.5%) of responded to at least one text message and replied to an average of 12.5 (SD = 3.45) texts. Response rates for each intervention arm are further illustrated in Table 2. Dropout over time was more pronounced in the two treatment intervention groups: the number of responding patients between the first and last week decreased by 14.0% in the acupuncture group, 12.6% in the counselling group and 1.9% in the usual care group.

**Table 2:** **R-SMS-DS response rates**

	Acupuncture n = 214		Counselling n = 207		Usual care n = 106		Total n = 527	
Number of patients responding to any text	206	96.3%	194	93.7%	98	92.5%	498	94.5%
Number of patients responding at Week 2	182	85.0%	167	80.7%	89	84.0%	438	83.1%
Number of patients responding at Week 16	152	71.0%	141	68.1%	87	82.1%	380	72.1%
Mean number of responses of responding patients	12.5	SD = 3.23	11.9	SD = 4.09	13.6	SD = 1.95	12.5	SD = 3.45

#### Participant comments

Verbal feedback, received through general communication, indicated that text messages were highly valued by participants, as a form of contact with the research team. Although instructed to reply only with a single digit, SMS responses frequently contained messages of gratitude. The overall acceptability of the SMS system to trial participants was also supported by a number of specific comments written in follow-up questionnaires. Hence:

*“As to the study itself, the ability to respond via text message has been excellent – very easy to respond to and very convenient.”*

(ID 1109)

*“The study overall helped me so much. It was a lifeline…..The most useful things were: 1) The texts rating 0-9? < Really nice!; 2) The questionnaires; 3) The excellent counselling sessions. Thank you!!”*

(ID 1170)

Whilst not a major problem, verbal feedback received from a small number of participants indicated some confusion regarding the response format. This was echoed in the written comments of a single participant:

*“I have been a little concerned about the texts, not sure if I was going the right way with the numbers. It was supposed to show an improvement – I hope it did!”*

(ID 1694)

In addition, narratives from several participants described the positive impact of answering questions and general contact with the research team, especially in terms of self-reflection and combatting feelings of isolation. Hence the following participants, both allocated to usual care alone, noted:

*“Filling the questionnaires in has been helpful as I have had to think about how I have felt so that I could answer the questions (my preference is to try not to think about anything and pretend there isn’t a problem)”.*

(ID 1145)

*“This study has helped me to realise things about myself. The care + concern given by the team when at my lowest was key to keeping me alive. I thank you for that.”*

(ID 1159)

### Validity

#### Score distribution

**Figure 2:**
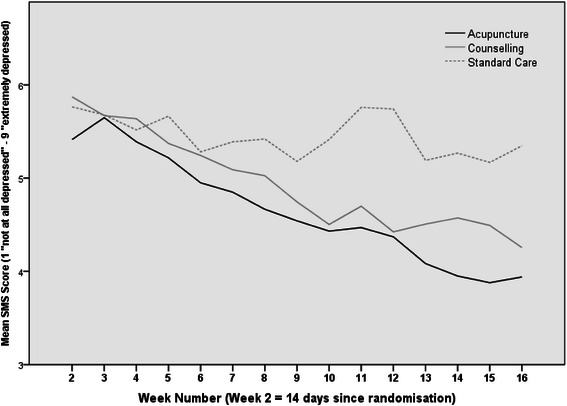
**Mean unadjusted (R-SMS-DS) text scores by trial arm (1 = not at all depressed, 9 = extremely depressed).**

Text responses contained the full range of scores from 1 to 9 and tended to be normally distributed (using all valid texts: n = 6137, mean = 5.0, SD = 2.18, Median = 5.0, Interquartile Range: 3.0-7.0). The majority of responses constituted whole numbers; only 1.6% were half scores between values. Unadjusted mean weekly text scores for each trial arm are presented in Table 3 and Figure 2. Over the 15 week study period, outcomes as reported by these depression scores generally improved for all patients.

**Table 3:** **Unadjusted mean R-SMS-DS text scores by trial arm**

Week	Acupuncture			Counselling			Usual care		
	n	Mean	SD	n	Mean	SD	n	Mean	SD
2	180	5.4	(1.57)	163	5.9	(1.87)	87	5.8	(1.70)
3	182	5.6	(1.83)	159	5.7	(2.01)	90	5.7	(1.88)
4	183	5.4	(1.82)	157	5.6	(2.09)	89	5.5	(2.08)
5	187	5.2	(1.89)	156	5.4	(2.05)	94	5.7	(1.99)
6	177	4.9	(1.92)	152	5.2	(2.09)	87	5.3	(2.01)
7	176	4.8	(1.96)	151	5.1	(2.13)	90	5.4	(2.08)
8	169	4.7	(1.98)	155	5.0	(2.22)	87	5.4	(2.15)
9	169	4.5	(2.14)	149	4.7	(2.29)	92	5.2	(2.22)
10	169	4.4	(2.27)	154	4.5	(2.24)	87	5.4	(2.20)
11	163	4.5	(2.35)	146	4.7	(2.29)	87	5.8	(2.01)
12	166	4.4	(2.19)	150	4.4	(2.28)	87	5.7	(1.94)
13	167	4.1	(2.20)	144	4.5	(2.40)	89	5.2	(2.09)
14	161	4.0	(2.09)	151	4.6	(2.37)	82	5.3	(2.10)
15	152	3.9	(2.11)	142	4.5	(2.46)	86	5.2	(2.31)
16	151	3.9	(2.31)	137	4.3	(2.35)	85	5.3	(2.28)

#### Concurrent validity

At week 16 post randomisation, 220 participants (63.6%) responded to the weekly depression text within 6 days of completing the PHQ-9 paper questionnaire. The two measures were moderately correlated at that point (Kendall’s tau-b = 0.57, p < 0.0001). Table 4 shows that the highest correlations between SMS depression scores and individual PHQ-9 items were seen for item 1 (*Little interest or pleasure in doing things*), item 2 (*Feeling down, depressed or hopeless*) and item 6 (*Feeling bad about yourself*). In comparison, the association between the validated PHQ-9 and BDI-II instruments was tau-b = 0.63 at baseline (n = 1408 patients screened for the ACUDep trial, p < 0.0001) and tau-b = 0.66 at 12 months (n = 548 patients in ACUDep follow-up, p < 0.0001).

**Table 4:** **Correlation between depression assessment tools (all p < 0.0001)**

Time	Assessment tool 1	Assessment tool 2	N	Kendall’s tau-b
**Baseline**	PHQ-9	BDI-II	1408^1^	0.6294
**3 months**	R-SMS-DS	PHQ-9 Total	220^2^	0.5704
	R-SMS-DS	PHQ-9 item 1	219^2^	0.5279
	R-SMS-DS	PHQ-9 item 2	220^2^	0.5316
	R-SMS-DS	PHQ-9 item 3	219^2^	0.3967
	R-SMS-DS	PHQ-9 item 4	219^2^	0.4206
	R-SMS-DS	PHQ-9 item 5	218^2^	0.4319
	R-SMS-DS	PHQ-9 item 6	219^2^	0.5673
	R-SMS-DS	PHQ-9 item 7	220^2^	0.4213
	R-SMS-DS	PHQ-9 item 8	219^2^	0.3786
	R-SMS-DS	PHQ-9 item 9	220^2^	0.4166
**12 months**	PHQ-9	BDI-II	548^3^	0.6632

^1^All patients screened at baseline.

^2^Patients in the SMS sub-study with text replies received within +/− 6 days of date of PHQ-9 completion.

^3^All patients in the ACUDep trial with response data.

### Utility

Figure 2 illustrates that scores decreased to a greater extent in the acupuncture and counselling groups (1.5 and 1.6 score points respectively) compared to the usual care group (0.5 score points) over the 15 week study period, mirroring findings from the main ACUDep trial analysis [22]. The trajectories appeared comparable between responders in the acupuncture and counselling groups.

The linear mixed model predicting SMS depression scores over 15 weeks (adjusting for baseline PHQ-9) revealed significant fixed effects of trial arm (F_2_ = 4.99, p = 0.007), time (F_14_ = 8.78, p < .001) and arm by time interaction (F_28_ = 1.78, p = 0.007). The interaction confirmed that depression trajectories over time differed between trial arms. Individual contrasts of trial arm at each week showed that additional improvements for acupuncture (difference of −0.77 score points compared to usual care) and counselling (difference of −0.82 score points compared to usual care) became significant from 10 weeks after randomisation onwards and increased until the end of the texting follow-up period (see Table 5 for adjusted means and group differences).

**Table 5:** **Linear mixed model** ^**1**^ **: adjusted means and effect of trial arm at each time point**

Time	Acupuncture		Counselling		Usual care		Effect of trial arm		
Week	Mean	SE	Mean	SE	Mean	SE	F	df	p
2	5.56	0.136	5.73	0.143	5.74	0.195	0.48	2	.621
3	5.76	0.134	5.56	0.142	5.60	0.191	0.54	2	.582
4	5.49	0.133	5.54	0.142	5.44	0.191	0.10	2	.909
5	5.26	0.132	5.30	0.142	5.57	0.188	0.98	2	.375
6	5.10	0.135	5.18	0.144	5.25	0.193	0.22	2	.806
7	4.95	0.137	5.03	0.146	5.31	0.193	1.23	2	.292
8	4.80	0.141	4.95	0.147	5.35	0.198	2.60	2	.074
9	4.78	0.144	4.74	0.152	5.15	0.199	1.56	2	.211
10	4.62	0.147	4.57	0.154	5.39	0.207	5.91	2	.003
11	4.67	0.153	4.69	0.161	5.67	0.213	8.55	2	.000
12	4.60	0.157	4.47	0.165	5.64	0.219	10.08	2	.000
13	4.36	0.162	4.62	0.172	5.20	0.225	4.60	2	.010
14	4.21	0.169	4.64	0.176	5.10	0.238	4.82	2	.008
15	4.18	0.177	4.53	0.185	5.11	0.243	4.78	2	.008
16	4.18	0.184	4.39	0.193	5.24	0.253	5.93	2	.003

^1^Model definition: SMS score over 15 weeks predicted by trial arm, time, arm x time interaction, adjusting for baseline PHQ-9.

**Figure 3:**
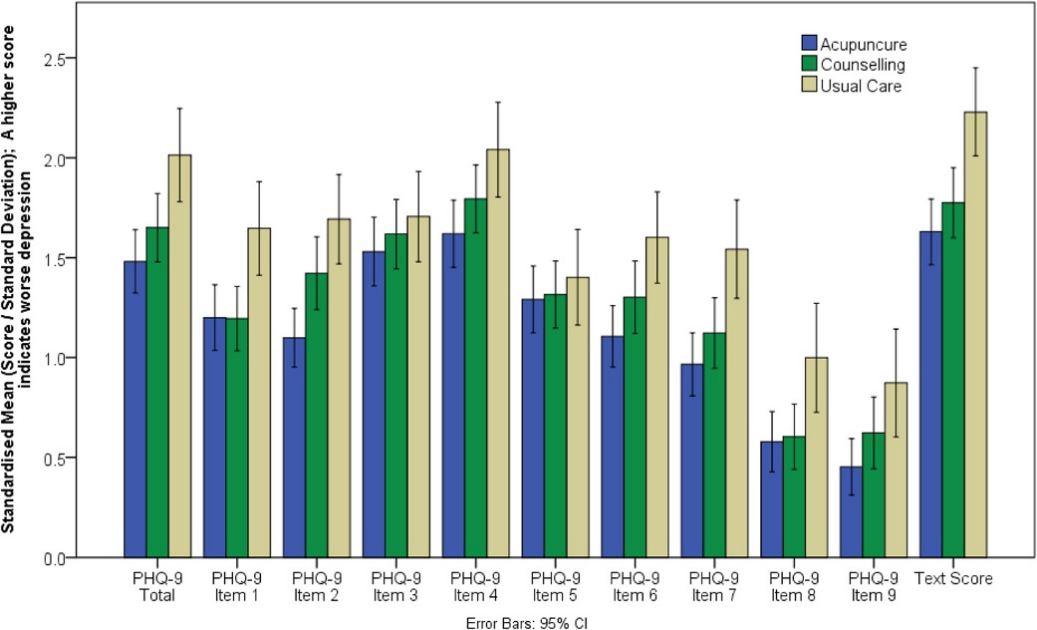
**Mean standardised outcomes for the PHQ-9 and R-SMS-DS text score at three months (SMS consenters only).**

As regards responsiveness, Figure 3 shows the unadjusted standardised outcome means for the PHQ-9 total, each individual PHQ-9 item, and the SMS text score by trial arm for patients who consented to take part in the SMS sub-study. Standardised scores, shown in Figure 3, are the score divided by the standard deviation, which are also provided in Table 6. Table 7 gives resulting standard effect sizes. This analysis showed that, when compared against usual care alone, the standardised mean difference in observed depression outcomes for (a) acupuncture and (b) counselling groups was greater for the R-SMS-DS (Effect sizes = 0.59 and 0.46 respectively) than for all but one individual item of the PHQ-9, and indeed total PHQ-9 scores (Effect sizes = 0.53 and 0.29 respectively). This finding suggests that overall the single item SMS depression scale was more sensitive in detecting changes resulting from treatment than the PHQ-9, further supporting its utility as a depression outcome measure.

**Table 6:** **Standardised mean scores (Score/Standard deviation) by outcome measure and treatment arm at 3 months**

Outcome measure	Acupuncture		Counselling		Usual care	
	Mean	SD	Mean	SD	Mean	SD
PHQ-9 Q1	1.18	1.007	1.28	.986	1.59	.989
PHQ-9 Q2	1.10	.915	1.46	1.035	1.70	.962
PHQ-9 Q3	1.50	1.022	1.61	.990	1.75	.984
PHQ-9 Q4	1.62	1.007	1.84	.969	2.05	.995
PHQ-9 Q5	1.27	1.019	1.39	.981	1.38	.990
PHQ-9 Q6	1.12	.924	1.37	1.058	1.56	.968
PHQ-9 Q7	0.95	.937	1.15	.966	1.52	1.076
PHQ-9 Q8	0.60	.946	0.64	.926	0.99	1.146
PHQ-9 Q9	0.46	.889	0.62	1.025	0.81	1.127
PHQ-9 Total	1.47	.977	1.70	.975	2.00	1.007
R-SMS-DS Week 16	1.66	.973	1.80	.992	2.26	.960

**Table 7:** **Score differences and standard effect sizes for treatment arm comparisons at 3 months for participants in the SMS sub-study**

	Acupuncture vs usual care				Counselling vs usual care				Acupuncture vs counselling			
Outcome	N	Score diff	SD*	Standard effect size	N	Score diff	SD*	Standard effect size	N	Score diff	SD*	Standard effect size
PHQ-9 Total	272	−3.40	6.45	0.53	258	−1.90	6.45	0.29	342	−1.50	6.45	0.23
PHQ-9 Item 1	271	−0.36	0.87	0.42	257	−0.28	0.87	0.32	342	−0.09	0.87	0.10
PHQ-9 Item 2	271	−0.54	0.91	0.60	257	−0.22	0.91	0.24	340	−0.33	0.91	0.36
PHQ-9 Item 3	271	−0.26	1.03	0.25	256	−0.14	1.03	0.14	341	−0.12	1.03	0.11
PHQ-9 Item 4	270	−0.42	0.96	0.43	258	−0.20	0.96	0.21	340	−0.22	0.96	0.23
PHQ-9 Item 5	270	−0.13	1.08	0.12	257	0.01	1.08	0.00	341	−0.13	1.08	0.12
PHQ-9 Item 6	271	−0.45	1.03	0.44	258	−0.20	1.03	0.19	341	−0.25	1.03	0.24
PHQ-9 Item 7	272	−0.56	0.99	0.57	258	−0.37	0.99	0.37	342	−0.20	0.99	0.20
PHQ-9 Item 8	272	−0.36	0.92	0.40	257	−0.33	0.92	0.36	341	−0.04	0.92	0.04
PHQ-9 Item 9	272	−0.27	0.79	0.35	258	−0.15	0.79	0.19	342	−0.12	0.79	0.15
R-SMS-DS Week 16	236	−1.41	2.37	0.59	222	−1.09	2.37	0.46	288	−0.32	2.37	0.14

*Standard Deviation of total sample used for all comparisons.

## Discussion

### Principal findings

The results of this study demonstrate that use of an automated SMS system offered a feasible, acceptable, inexpensive and valid method of measuring change in depression, for the purposes of clinical research.

This system was widely adopted as a means of reporting changes in mood, on a weekly basis, by patients with moderate to severe depression who had volunteered as participants in a larger randomised controlled trial studying the comparative therapeutic effectiveness of acupuncture, counselling, and usual GP care. Participants reported that they liked receiving and responding to regular text messages which asked about their mood. In conjunction with other means of communication, this offered participants an opportunity to reflect, to feel cared for, and helped to combat loneliness.

Use of the automated SMS system as a means of data collection amongst patients with moderate to severe depression was not without problems however. Responses required regular monitoring, as some participants assumed that their texts would be read by a member of the research team upon receipt, and therefore tried to use the system to convey other information or requests. In a few cases, responses received via this system indicated impending personal risk or raised other serious concerns regarding participant welfare, leading to the involvement of specialist mental health services.

Other problems associated with our use of the automated SMS system described included the occurrence of a technical error, which resulted in loss of data. Besides system reliability, the use of third parties to distribute and gather text messages poses questions regarding data protection, which may require further clarification. Rather than providing only numerical data, as requested, participants in this study sometimes sent unsolicited non-numerical information to the research team via text message. Therefore, in addition to ensuring that appropriate security arrangements are in place, we recommend that all research participants are informed that any information they send via SMS will be handled by a third parties, including both the SMS system provider and their mobile network operator. Systems can also be developed to reject non numerical responses, or trigger automatic alerts in response to messages containing any pre-specified words which may indicate risk of self-harm [21].

As regards expense, text messaging cost approximately 52 to 53 pence per valid response, which excluded additional resources involved in monitoring incoming texts. This compares very favourably with other data collection methods. For example, current UK postage costs involved in sending and receiving just one questionnaire generally exceed £1, which alone does not account for additional printing and data management costs, reminder letters, or payment of licence fees for using instruments such as the BDI-II. Importantly, in our reporting of the development, content, and validation of the single item SMS depression rating scale, we place this instrument (the R-SMS-DS) in the public domain to be used freely, conditional only upon appropriate acknowledgement of authorship in any published work. Costs associated with gathering data via SMS may also be reduced further by providing study participants with access to a ‘Free text’ service, instead of sending each participant £5 in advance, as happen in the present study, although this in turn might actually serve as less of an incentive for participants to reply.

Comparison of responses for the R-SMS-DS with those for the PHQ-9 at three months demonstrated a moderate to high degree of convergence between instruments, thereby offering supportive evidence for construct validity. Given the simplicity of this single item nine-point depression rating scale, and mode of administration, this is encouraging, especially when one considers that the observed degree of association between responses for the BDI-II and the PHQ-9, both psychometrically robust depressions outcome measures, was only marginally greater.

Additional evidence for the utility and responsiveness of the SMS depression rating scale as a valid depression outcome measure was provided by our ability to plot and identify statistically significant treatment effects emerging from both acupuncture and counselling, when compared to usual care alone, just ten weeks after randomisation (typically after eight consecutive treatment sessions), which were later detected using the PHQ-9 on questionnaires at three months. Moreover, the R-SMS-DS outperformed the PHQ-9 in terms of its sensitivity for measuring changes in depressions resulting from treatment.

### Strengths and limitations

The present study offers a unique insight into the probable future use of text messaging as a valid data collection tool for clinical research on depression. It is almost certainly the largest study of its kind, involving several hundred participants. It also describes the development and validation of a new outcome measure for depression, which lends itself more readily to frequent data collection, and appears somewhat more responsive than other established measurement approaches. One limitation of the study is that we were unable to estimate the convergent validity between the SMS depression rating scale and the PHQ-9 at baseline, due to differences in the timing of administration. Similarly, further evidence relating the construct validity of the R-SMS-DS might have been gathered had we taken the opportunity to administer the BDI-II at three month follow up.

### Comparison with previous research findings

Previous research concerning the use of text messaging as a data collection tool for the measurement of change in depression is extremely limited. However, the present findings appear to confirm wider findings on the popularity and acceptability of text messaging amongst participants in clinical research, and advantage over other data collection methods for regularly capturing simple self-rated item scores over time as additional study outcomes, with minimal inconvenience to study participants.

### Recommendations for research and practice

More research is recommended to replicate and build upon the present study. Nevertheless, given the findings of this study, we recommend use of the R-SMS-DS by researchers and clinicians in the field of mental health, who may wish to include it alongside other relevant outcome measures, for the purpose of monitoring and plotting changes in depression over time and comparing the effectiveness of different treatments. Caution should be urged however, in ensuring that adequate procedures are put in place to monitor the content of incoming texts and, where relevant, notify participants in advance that any personal information they provide will be handled by a third party. Future research might also consider the possibility that regular collection of data using this outcome measure could itself have a therapeutic effect, as indicated by feedback from one of the participants in this study, but which unfortunately this study was not designed or powered to detect.

## Conclusions

The findings of this study demonstrate that automated text messaging is a feasible, inexpensive and acceptable method of collecting clinical outcome data on depression. It also enables researchers to actively monitor and plot changes in depression on a much frequent basis than traditional data collection methods. The SMS item and corresponding nine-point depression rating scale developed in this study showed good evidence for construct validity, when compared with other depression outcome measures. Findings from this study also indicated that overall the SMS instrument was more sensitive than the PHQ-9 in measuring treatment effects arising from the provision of acupuncture and counselling, being successfully employed to identify the presence of a statistically significant treatment effects at an earlier stage than that of a standard postal questionnaires. Nevertheless, such systems require active monitoring, and researchers will need to be alert for rare but disturbing responses from people who may be at immediate risk of harm to themselves. Accompanying this are relevant ethical and legal responsibilities which require consideration. As indicated by one of the participants in this study, appropriate collaboration between researchers and clinicians in identifying and handling such risks also has the potential to save lives.

## Abbreviations

ACUDep: Acupuncture, counselling, and usual care for depression
BDI-II: Beck Depression Inventory-II
PHQ-9: Patient Health Questionnaire 9
R-SMS-DS: Richmond et al. short mesage service depression scale
RCT: Randomised controlled trial
